# Comparison of Radiation-Induced Bystander Effect in QU-DB Cells after Acute and Fractionated Irradiation: An In Vitro Study

**DOI:** 10.22074/cellj.2016.4562

**Published:** 2016-08-24

**Authors:** Shokouhozaman Soleymanifard, Mohammad Taghi Bahreyni Toossi, Roghayeh Kamran Samani, Shokoufeh Mohebbi

**Affiliations:** 1 Medical Physics Research Center, Mashhad University of Medical Sciences, Mashhad, Iran; 2 Department of Medical Physics, School of Medicine, Mashhad University of Medical Sciences, Mashhad, Iran; 3 Department of Medical Physics, Omid Hospital, Mashhad University of Medical Sciences, Mashhad, Iran

**Keywords:** Bystander Effects, Dose Fractionation, Micronucleus Assay, Radiation

## Abstract

**Objective:**

Radiation effects induced in non-irradiated cells are termed radiation-induced bystander effects (RIBE). The present study intends to examine the RIBE response of QU-DB bystander cells to first, second and third radiation fractions and compare their cumulative outcome with an equal, single acute dose.

**Materials and Methods:**

This experimental study irradiated three groups of target cells for one, two and three times with^60^Co gamma rays. One hour after irradiation, we transferred their culture media to non-irradiated (bystander) cells. We used the cytokinesis block micronucleus assay to evaluate RIBE response in the bystander cells. The numbers of micronuclei generated in bystander cells were determined.

**Results:**

RIBE response to single acute doses increased up to 4 Gy, then decreased, and finally at the 8 Gy dose disappeared. The second and third fractions induced RIBE in bystander cells, except when RIBE reached to the maximum level at the first fraction. We split the 4 Gy acute dose into two fractions, which decreased the RIBE response. However, fractionation of 6 Gy (into two fractions of 3 Gy or three fractions of 2 Gy) had no effect on RIBE response. When we split the 8 Gy acute dose into two fractions we observed RIBE, which had disappeared following the single 8 Gy dose.

**Conclusion:**

The impact of dose fractionation on RIBE induced in QU-DB cells de- pended on the RIBE dose-response relationship. Where RIBE increased proportion- ally with the dose, fractionation reduced the RIBE response. In contrast, at high dos- es where RIBE decreased proportionally with the dose, fractionation either did not change RIBE (at 6 Gy) or increased it (at 8 Gy).

## Introduction

Radiation-induced bystander effect (RIBE) occurs when non-irradiated cells receive molecular signals produced by irradiated cells. This leads to radiation injuries in non-irradiated cells. Humans and other organisms may undergo irradiation a number of times; however, it is not clear whether RIBE is induced only once or can be induced as a result of subsequent irradiation. It is also unclear, whether the amount of RIBE damages induced by a single acute dose are equal, less, or more than the RIBE damages induced by an equal fractionated dose. Few researchers have investigated this subject. Widel divided a 1.5 Gy dose into three equal fractions. The results showed that all three fractions induced RIBE damages in bystander cells. However, when the same procedure was performed at 6 Gy, only the first fraction influenced the bystander cells (1). Mothersill and Seymour (2) compared the outcome of a specified acute dose with an equal fractionated dose. They observed that the fractionated dose was more toxic than the equal acute dose. In a previous study we evaluated the extent of damages induced in MRC5 bystander cells as a result of acute or fractionated radiation doses (two subsequent fractions). The results were evident that the second fraction did not induce RIBE in bystander cells (3). The RIBE response in MRC5 cells was constant or independent of the radiation dose. Therefore we attributed the lack of RIBE in the second fraction to the constant response of MRC5 cells to RIBE. We predicted that the QU-DB cell line might be affected by subsequent dose fractions, as their response to RIBE increased at higher radiation doses (3). Therefore, the present *in vitro* study intended to examine the RIBE response of QU-DB cells to first, second and third radiation dose fractions, and compare their cumulative outcome with an equal single acute dose. 

## Materials and Methods

### Cell culture

The QU-DB cell line was supplied by Pasteur Institute, Tehran, Iran, and applied in this experimental study. The cell line was established from a patient with large cell anaplastic lung carcinoma in 1986 (4). The cells were grown in RPMI-1640 medium (Biosera, England) supplemented with 10% fetal bovine serum (Biosera, England), 100 U/ml penicillin (Biosera, England), and 100 μg/ml streptomycin (Biosera, England). The cultures were maintained at 37˚Cin a humidified atmosphere of 95% air and 5% CO_2_.

### Irradiation

Sub-confluent cells were trypsinized and cultured in 10 cm^2^ flasks. One day later, we placed the flasks on a water phantom (30×30×10 cm), after which they were irradiated with gamma rays emitted from a^60^Co Teletherapy Unit (Theratron, Phoenix model, average dose-rate: 71.89 Gy/minute) at room temperature. The field size was 15×15 cm^2^ and source to medium surface was 80 cm. The height of the culture media in the flasks was 5 mm, which indicated that cells attached to the bottom of the flasks received a maximum dosage of^60^Co gamma rays.

### Medium transfer

We applied the medium transfer technique to induce bystander effect in the non-irradiated cells. After irradiation, target cells were incubated for one hour after which their culture medium was extracted, filtered through a 0.22 µm acetate cellulose filter (Orange Scientific, Belgium), and finally transferred to the bystander cells. 

### Experimental groups

We defined two main groups: acute and fractionated dose. The bystander cells which received medium from the cells irradiated once were considered the acute dose group. The acute subgroups (1×2 Gy, 1×4 Gy, 1×6 Gy, and 1×8 Gy) received conditioned medium from the cells irradiated with 2, 4, 6, and 8 Gy. The fractionated groups received conditioned medium from the cells irradiated two or three times at half or one third of the acute dose with a time interval between fractions equal to 6 hours. After each dose fraction we extracted the conditioned medium from the irradiated cells and transferred it to the bystander cells. Subgroups that received conditioned medium from cells irradiated twice with 2, 3, and 4 Gy were labeled as: 2×2 Gy, 2×3 Gy, and 2×4 Gy. The 3×2 Gy subgroup received medium from cells irradiated three times with 2 Gy. Two control groups received medium from the acute and fractionated sham-irradiated cells. 

### Radiation-induced bystander effect response

We quantified the RIBE response by determining the numbers of bystander cells that contained micronuclei per 1000 binucleated cells (MNBN) and the Nuclear Division Cytotoxicity Index (NDCI) of the bystander cell groups. After the last medium transfer we added cytochalasin B (0.8 μg/ mL) to the bystander flasks, then the cytokinesis block micronucleus assay was applied. Cells in the flasks that contained cytochalasin B were incubated for 24 hours, then fixed three times with a 3:1 ratio of methanol acetic acid (Merck, Germany). Each time that a fresh mixture of methanol and acetic acid was added to the flasks, we incubated the cells at 4˚C for 20 minutes. Then, the fixator was removed and the cells were dried. Next, the cells were stained with 10% giemsa for 7 minutes. Finally, stained cells that attached to the bottoms of the flasks were scored by a light microscope (Olympus BH-2) at ×400 magnification. In order to determine the number of micronucleated cells, at least 1000 binucleated cells per flask were scored. NDCI is an appropriate parameter which determines the nucleus division status of cells in response to cellular toxicity. The number of mono-, bi-, and multinucleated cells, as well as apoptotic and necrotic cells were counted. We determined the NDCI according to the following equation, based on Fenech (5): 

$$\frac{\mathrm{NCDI}=\mathrm{AP}+\mathrm{Nec}+\mathrm{M1}+(2\times \mathrm{M2})+3\times \mathrm{M3})+4\times \mathrm{M4})}{N}$$

Where: 

AP=Number of apoptotic cells

Nec=Number of necrotic cells

M1-M4=Number of viable cells with one to four nuclei

N=Total number of cells scored (viable and nonviable) 

### Statistical analysis

The data were acquired based on at least six independent measurements for each subgroup. The Kolmogorov–Smirnov test showed normal data distribution. Therefore, we used the one-way analysis of variance, Tukey’s multiple comparison and Dunnett’s tests to compare the groups. 

### Results

**Fig.1:**
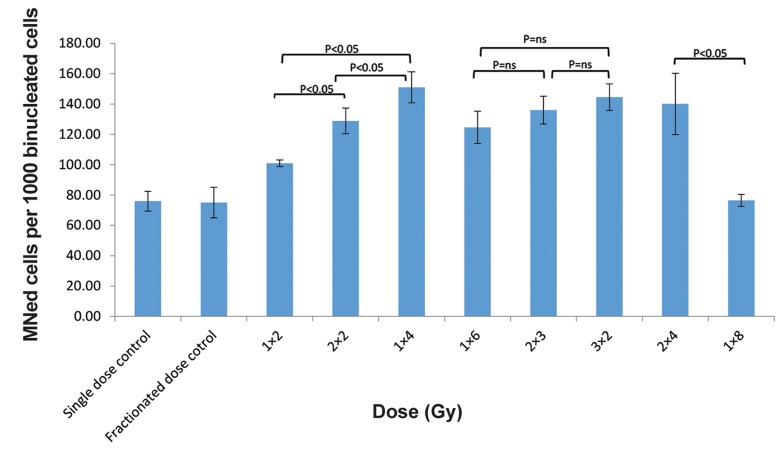
Mean number of micronucleated cells per 1000 binucleated cells (MNBN) in both single and fractionated dose groups. Error bars indicate the SEM for six independent experiments. ns; Non-significant.

Table 1 and Figure 1 show the frequency of MNBN cells in different subgroups. With the exception of the 1×8 Gy subgroup (P=1.000), all subgroups showed statistically significant differences compared with their control groups (P<0.05). 

We sought to determine whether RIBE was induced following the second and third fractions by comparing the 1×2 Gy, 2×2 Gy, and 1×4 Gy subgroups to the 2×2 Gy, 3×2 Gy, and 2×4 Gy subgroups. There were statistically more MNBN cells counted in the 2×2 Gy subgroup compared to the 1×2 Gy subgroup (P<0.001). The number of MNBN cells in the 3×2 Gy subgroup was more than the MNBN cells counted in the 2×2 Gy fractionated subgroup (P<0.05). There was no significant difference between MNBNs counted in the 2×4 Gy and 1×4 Gy subgroups (P=0.102). 

The above results revealed that RIBE was induced following the second and third fractions; therefore, we compared the acute and fractionated subgroups (at the same total dose) in order to determine whether fractionation of a specified dose decreased or increased the RIBE response. The 1×4 Gy and 1×8 Gy subgroups were compared with the 2×2 Gy and 2×4 Gy subgroups, respectively. Statistical analysis revealed that a higher number of MNBN cells formed following the acute dose of 4 Gy (1×4 Gy) compared with the fractionated dose (2×2 Gy, P<0.001). However the acute dose of 8 Gy (1×8 Gy) was less effective than the corresponding fractionated dose (2×4 Gy, P<0.001). A comparison of the 1×6 Gy subgroup with the 2×3 Gy and 3×2 Gy subgroups showed no significant difference between the three subgroups (P=0.082). 

Table 2 shows the NDCI of the groups. The NDCI was not statistically different for all subgroups (P=0.059). 

**Table 1:** Mean number of micronucleated cells per 1000 binucleated cells (MNBN) in bystander cell groups


Group	Subgroup	MNBN (Mean ± SD)	Range	P value

Single dose	0 (Single dose control)	76 ± 6.54	65-83	-
	1×2 Gy	101 ± 2.19	98-104	0.004
	1×4 Gy	151 ± 10.19	138-164	0.000
	1×6 Gy	124.67 ± 20.23	100-150	0.000
	1×8 Gy	76.5 ± 3.93	71-81	1.000
Fractionated dose	0 (Fractionated control)	75.3 ± 8.11	67-89	-
	2×2 Gy	128.83 ± 8.47	117-142	0.000
	2×3 Gy	136 ± 8.76	128-147	0.000
	3×2 Gy	144.5 ± 10.67	131-156	0.000
	2×4 Gy	140.5 ± 9.16	132-157	0.000


P value indicates statistically significant difference between subgroups and their controls.

**Table 2:** Nuclear Division Cytotoxicity Index (NDCI) of single and fractionated dose groups


	0 (Fractionated dose)	0 (Single dose)	1×2 Gy	2×2 Gy	1×4 Gy	2×3 Gy	3×2 Gy	1×6 Gy	2×4 Gy	1×8 Gy

NDCI	1.45	1.44	1.39	1.38	1.45	1.40	1.41	1.51	1.38	1.45


## Discussion

The results indicated that the second and third fractions (2×2 Gy and 3×2 Gy subgroups) affected the QU-DB cells; however, when RIBE reached a maximum level at the first fraction (in the 2×4 Gy group), the second fraction could not increase the RIBE level. We observed that the impact of dose fractionation on the RIBE response depended on the RIBE dose-response relationship. Where RIBE increased proportionally with the dose (below 4 Gy), fractionation reduced the RIBE response. While at high doses where RIBE decreased proportionally with the dose, fractionation either did not change RIBE (at 6 Gy) or increased it (at 8 Gy).

Analysis of the numbers of MNBN cells in the acute radiation groups has shown that below 4 Gy RIBE increased proportionally in a dose-dependent manner whereas above 4 Gy, RIBE decreased despite the dose increase. The results of the micronucleus assay depend on the cell replication status, hence we have determined the NDCI from all groups in order to ascertain whether the reduction in MNBN was attributed to delays in cell replication. The results showed no significant difference between the groups and their corresponding controls (P>0.05). Hence, MNBN reduction was not due to a replication delay; rather, the delay was an actual RIBE reduction. The results of a parallel, nonpublished study (researcher communication) that researched other end points confirmed the dose-response curve observed in QU-DB cells. Other researchers observed decreased RIBE at high doses (1,6,9). This finding was interpreted as a result of a stimulated repair mechanism (6) or negative feedback activated in bystander cells (8). Disappearance of RIBE in the 1×8 Gy group might be attributed to such mechanisms. These mechanisms were less effective in the 1×6 Gy group compared with the 1×8 Gy group. 

In order to answer whether RIBE was induced in all fractions or only in the first one, we compared the 1×2 Gy, 2×2 Gy and 3×2 Gy subgroups with each other. In addition, the 1×4 Gy was compared with the 2×4 Gy subgroup. The results indicated that when the dose per fraction was below 4 Gy, the second and third fractions affected QU-DB bystander cells. There was a higher MNBN frequency observed in the 2×2 Gy subgroup compared to the 1×2 Gy subgroup, as well as in the 3×2 Gy subgroup compared with the 2×2 Gy subgroup. At 4 Gy, the second fraction could not increase the RIBE level. The frequency of MNBN cells in the 2×4 Gy and 1×4 Gy subgroups was equal. The reason might be that RIBE at 4 Gy reached a maximum level; therefore, subsequent irradiation could not affect the bystander cells and increase RIBE above the maximum level. In other words, the second and third fractions could not affect QUDB bystander cells unless RIBE had not reached its maximum level in the first fraction. These results supported our previous results (3). We observed a constant RIBE level in the MRC5 cells and the second fraction had no effect on bystander cells. It was suggested that the constant RIBE level in MRC5 cells was a saturated/maximum level, which consequently could not be increased by a subsequent fraction. 

The level of RIBE in acute irradiation and corresponding fractionated protocols was determined by comparing the 1×4 Gy and 1×8 Gy acute subgroups with the 2×2 Gy and 2×4 Gy fractionated subgroups. We also compared the 1×6 Gy acute subgroup with the 2×3 Gy and 3×2 Gy fractionated subgroups. MNBN frequency in the 2×2 Gy subgroup was less than the 1×4 Gy subgroup which indicated that at the total dose of 4 Gy, fractionation decreased the damage induced in bystander cells. In contrast, we observed a higher MNBN frequency of the 2×4 Gy fractionated subgroup compared to the 1×8 Gy acute irradiation subgroup. At the total dose of 8 Gy, the resulted indicated that fractionation increased RIBE. There was no difference between the 6 Gy acute irradiation and fractionated subgroups. 

The MNBN frequency in the 1×6 Gy subgroup was similar to the 3×2 Gy and 2×3 Gy subgroups. To interpret the results, we should consider the two different regions of RIBE dose-response curve. The region below the 4 Gy dose had an incremental response; therefore, at 2 Gy RIBE did not saturate or did not reach the maximum level. Hence, it was possible for the second fraction to induce additional damage in bystander cells. As there was a gap between the fractions, bystander cells might repair some of their damage and consequently RIBE that resulted from two fractions was less than the RIBE of the 4 Gy acute irradiation dose. The increase of RIBE in the fractionated 8 Gy subgroup might be explained based on the stimulated repair mechanism or negative feedback hypotheses proposed by Gow et al. (8) and Makonis et al. (6), respectively. At a high dose per fraction (8 Gy), a stimulated repair mechanism or negative feedback appeared in bystander cells and prevented RIBE damage. However, at a lower dose (4 Gy per fraction) the above mechanisms were silent and RIBE damages were induced in bystander cells. These results were similar to our previous study of MRC5 cells. IBE disappeared when MRC5 cells received conditioned medium from 4 Gy irradiated QU-DB cells (3). However when we divided the 4 Gy dose into two fractions, RIBE revived. 

It was interpreted that fractionation prevented the stimulated repair mechanism/negative feedback and caused some damage in MRC5 bystander cells. At the 6 Gy dose, we observed no differences between acute and fractionated irradiation. The reason might be explained as follows. In groups 2×3 Gy and 3×2 Gy there was a gap between fractions which let bystander cells repair their damage. 

On the other hand, above 4 Gy stimulated repair or negative feedback decreased the damages induced by the 6 Gy acute irradiation. Therefore, it could be suggested that the decrease in RIBE due to a gap between fractions was equal to the amount of RIBE that decreased due to stimulated repair/negative feedback. In summary it could be suggested that the effect of radiation fractionation in QU-DB cells depended on the radiation dose. 

The 4 Gy dose is a critical dose which induces maximum RIBE in QU-DB cells. Fractionation of a radiation dose located in the incremental region of the dose-response curve (below 4 Gy) may cause a reduction in bystander damages. Above 4 Gy, stimulated repair/negative feedback due to a relatively high dose may compete with cellular repair induced as a result of a gap between the fractions. 

Very few studies have investigated the effects of radiation fractionation on RIBE, with versatile results. Mothersill and Seymour (2) measured the effects of two fractions of exposure to conditioned medium harvested from target cells. They also studied the cumulative effect of direct irradiation in one session and exposure to conditioned medium in the subsequent session or vice versa. They suggested the fractionated dose was more toxic than the equal acute dose in bystander cells, and the mixture of direct irradiation and exposure to conditioned medium might eliminate the bystander effect. Ilnytskyy et al. (10) studied the impact of dose fractionation on RIBE in an *in vivo* study. They irradiated two groups of mice. The first group received whole body irradiation, whereas in the second group only the skull was irradiated. The spleen was considered to have direct irradiation in the first group and a bystander organ in the second group. They measured microRNAome and DNA methylation in the spleen. Results showed dose-fractionation decreased the changes of DNA methylation in the directly irradiated spleen. However, epigenetic changes induced in the bystander spleen were permanent and did not decrease as a result of dose fractionation (10). In the above study the inefficacy of dose fractionation on RIBE in an animal model was observed. Widel measured the number of micronuclei in human melanoma cells. The results showed that when 1.5 Gy was divided into three fractions, all fractions induced micronuclei in bystander cells. However, when 6 Gy was divided into three fractions, only the first fraction induced a bystander effect. The second and third fractions were ineffective. These results were in line with our results which indicated the impact of fractionation on RIBE depended on the dose-range. 

The impact of dose rate and fractionation on RIBE has implications for carcinogenesis, radioprotection, and the refinement of radiotherapy (11). In terms of radioprotection dose rate may affect RIBE; in the field of radiotherapy RIBE may be affected by both dose rate and fractionation. In the present study the target cells have been irradiated with doses used in radiotherapy. The result may be used to discuss the impact of RIBE on different fractionation protocols in radiotherapy, where both normal and tumor cells can be affected by RIBE. When bystander signals are received by non-irradiated normal cells they may increase radiotherapy side effects. When they are received by tumor cells in the radiation field, they may enhance tumor cell killing. The results of this study have shown a direct relation between the impact of dose fractionation on RIBE to the total dose and the dose per fraction. Therefore, as different fractionation protocols are used in radiotherapy the outcome of RIBE may differ. The results of this study have been observed in QU-DB cells. Whether other tumor or normal cells have such a response should be determined in future studies. 

## Conclusion

The importance of RIBE in radiotherapy has been mentioned by some authors. On the other hand, as broadly fractionated protocols are applied in radiotherapy it is necessary to obtain complete knowledge about the impact of radiation fractionation on RIBE. In some cases such as grid therapy, stereotactic radiotherapy, brachytherapy, and hypofractionated protocols a relatively high dose is applied per fraction. It is probable the RIBE due to hypofractionated protocols performed in these modalities is different and influences their radiobiological outcomes. Consequently more studies should discuss this issue. Based on our results and other studies, it is predicted that the impact of dose fractionation on RIBE is dependent on cell type, dose per fraction, and total dose applied in a specified radiotherapy protocol. Therefore, it is suggested that a variety of normal/tumor cells and a broad range of radiation doses should be considered in new studies that deal with this issue. Of note, animal models are preferred to cell cultures which are either far from real radiotherapy conditions or are limited in relevance to the number of fractions that can be applied. 
